# Increased brain activation and functional connectivity after working memory training in patients with ischemic stroke: an fMRI study

**DOI:** 10.3389/fstro.2023.1189573

**Published:** 2023-09-28

**Authors:** Zhengwei Chen, Xiaoping Yun

**Affiliations:** ^1^Department of Neurology, The Second Affiliated Hospital of Xuzhou Medical University, Xuzhou, Jiangsu, China; ^2^Department of Rehabilitation Evaluation, China Rehabilitation Research Center, Beijing, China; ^3^Faculty of Rehabilitation, Capital Medical University, Beijing, China

**Keywords:** ischemic stroke, working memory training, cognitive rehabilitation, fMRI, brain activation, functional connectivity

## Abstract

**Objective:**

Working memory (WM) impairment is common in patients after a stroke. WM training (WMT) has been suggested as a way to improve cognitive function. However, the neural effects following WMT in stroke patients remain largely unclear. This study aimed to explore the behavioral changes and neural effects of WMT on patients with chronic ischemic stroke.

**Methods:**

Fifty first-ever ischemic stroke patients with WM deficits in the chronic stage were randomly assigned to either a 4-week WMT group or a control group. Verbal n-back, digital and spatial memory-span, Raven's standard progressive matrices, and the Stroop color-word test, as well as task-state and resting-state fMRI were assessed for all patients at baseline and after the intervention.

**Results:**

The WMT group showed improvements in WM, fluid intelligence, and attention after training. Additionally, the WMT group exhibited increased activation in the left middle frontal gyrus (MFG) and middle occipital gyrus after training. At baseline, all patients were impaired in their abilities to elevate activation in their WM network as a response to increasing WM load. However, in the WMT group, increased activation was observed in the left cerebellum anterior lobe, right cerebellum posterior lobe (CPL), and MFG in the 2-back vs. 1-back contrast after WMT. We also found increased functional connectivity between the left MFG and the left inferior parietal lobule (IPL), and between the bilateral IPL and the right CPL after training in the WMT group.

**Conclusion:**

Our study supported that WMT potentially improved WM capacity in ischemic stroke patients during the chronic stage, and that the training effects might transfer to fluid intelligence and attention ability. Our results also demonstrated that repeated WMT potentially increased brain activation and resting-state functional connectivity within the WM network in patients with ischemic stroke. These findings provided robust evidence to support WMT as an effective intervention to enhance cognitive rehabilitation and shed light on the functional neuroplasticity mechanism of WMT on cognitive recovery after ischemic stroke.

## Introduction

It is believed that one person dies from a stroke every 4 min (Benjamin et al., 2017). For stroke survivors, cognitive impairment is one of the common dysfunctions that results in a poorer quality of life and is one of the main reasons preventing patients from returning to family and society (Hochstenbach et al., 2001; Van Der Flier et al., 2018). It has been reported that the most common cognitive symptoms following stroke are memory loss (90%), attention deficit (82%), and executive dysfunction (75%) (Nakajima, 2006). Working memory (WM), an important part of cognitive function, refers to a memory system with limited ability to process and store information temporarily (Baddeley, 2003). It is now generally believed that there exist age-related changes in WM capacity in the normal population: verbal WM peaks at ~age 26 and visual-spatial WM peaks at ~age 18, and normal older adults suffer from WM capacity decreases (Hale et al., 2011; Swanson, 2017). WM is vital for tasks that involve the goal-oriented use of immediate memory, the storage and manipulation of recently assimilated information, and the conversion and scheduling of task priorities in multitasking situations (Redick and Lindsey, 2013; Kumar et al., 2016). The incidence of WM impairment among post-stroke patients is reported to be as high as 87.6% (Jaillard et al., 2009). WM deficits remain prominent in the chronic stage of stroke (Kant et al., 2014). Lesion studies have revealed that the fronto-parietal cortex is related to WM deficits in patients with stroke (Baldo and Dronkers, 2006; van Asselen et al., 2006). A meta-analysis and systematic review study suggested that patients with stroke showed decrements of moderate magnitude in both low-load and high-load WM tasks and in all subsystems of WM (including central executive, episodic buffer, visual-spatial sketchpad, and phonological loop) (Baddeley, 2012; Lugtmeijer et al., 2021). Deficits in the WM system may affect executive function along with shifting and inhibition, episodic memory formation and retrieval, general intelligence, and syntactic processing (Conway et al., 2003; Bergmann et al., 2013; Friedman and Miyake, 2017; Alatorre-Cruz et al., 2019).

While the conventional concept was that the WM capacity of adults was constant, subsequent studies have found that WM capacity could be improved through training. WM training (WMT) is a cognitive training method that focuses on WM tasks operated on a computer in an adaptive way. The difficulty of the training task can be adjusted to maximize the WM capacity of the trainee. The methods of WMT can be divided into WM span tasks (e.g., number forward and backward, spatial forward and backward), updating tasks (e.g., n-back), and complex WM tasks (e.g., verbal WM tasks combined with spatial WM tasks) according to the training tasks used. Studies have shown that WMT could significantly improve the WM ability of healthy subjects (Olesen et al., 2004), as well as the WM ability of acquired brain injury patients with WM disorder (Richter et al., 2018). In addition, it was suggested that the efficacy of WMT could be transferred to other untrained cognitive functions, such as attention (Jaeggi et al., 2008), fluid intelligence (Westerberg et al., 2007), reading (Loosli et al., 2011), inhibition (Holmes et al., 2009), and mathematics (Kelly et al., 2006).

It has been suggested that WMT is an effective cognitive rehabilitation strategy in stroke patients for improving individual WM capacity, as well as the performance of untrained WM tasks and tasks that show far-transfer effect (Peers et al., 2020; Eschweiler et al., 2021; Nikravesh et al., 2021). However, the neural effects following WMT in stroke patients remain largely unclear and have been poorly studied. Using the blood oxygenation level dependent (BOLD) technique, functional magnetic resonance imaging (fMRI) can accurately detect the brain regions that are closely related to individual WM, as well as the brain regions that show functional changes after WMT. Thus, the present study aimed to explore the behavior and neural effects of WMT on chronic stroke patients using neuropsychological scales and task- and resting-state fMRI. We hypothesized that patients with stroke in the present study might have improved WM capacity and altered brain activation and functional connectivity after the WMT.

## Materials and methods

### Subjects

Fifty ischemic stroke patients were recruited from the China Rehabilitation Research Center (CRRC) who met the following criteria: (1) right-handed, 18–65 years old, junior high school or above; (2) first-ever ischemic stroke; (3) cognitive function was normal before stroke; (4) stroke onset of more than 6 months; (5) stroke lesions located within the unilateral frontal or parietal cortex, as identified by CT or MRI; (6) Montreal Cognitive Assessment (MoCA) ≥20; (7) memory-span test was forward ≤ 6 and/or backward ≤ 5. Exclusion criteria were: (1) hemorrhagic stroke or having other underlying neurological diseases; (2) patients with psychiatric disorders, aphasia, unilateral neglect, agnosia, apraxia, hearing or visual impairments were excluded; (3) patients with MRI contraindications or presence of irremovable metallic substances affecting the quality of the scanned images were excluded; (4) patients with alcohol or drug abuse were excluded.

This clinical trial was registered at ClinicalTrials.gov, and the authors confirm that all ongoing and related trials for this intervention are registered; the registration number is NCT03012269. Ethical approval was given by the medical ethics committee of the CRRC (the approval number is NSFC-81272165) and all the participants signed informed consent forms before entering the study.

### Study design

**Figure 1:**
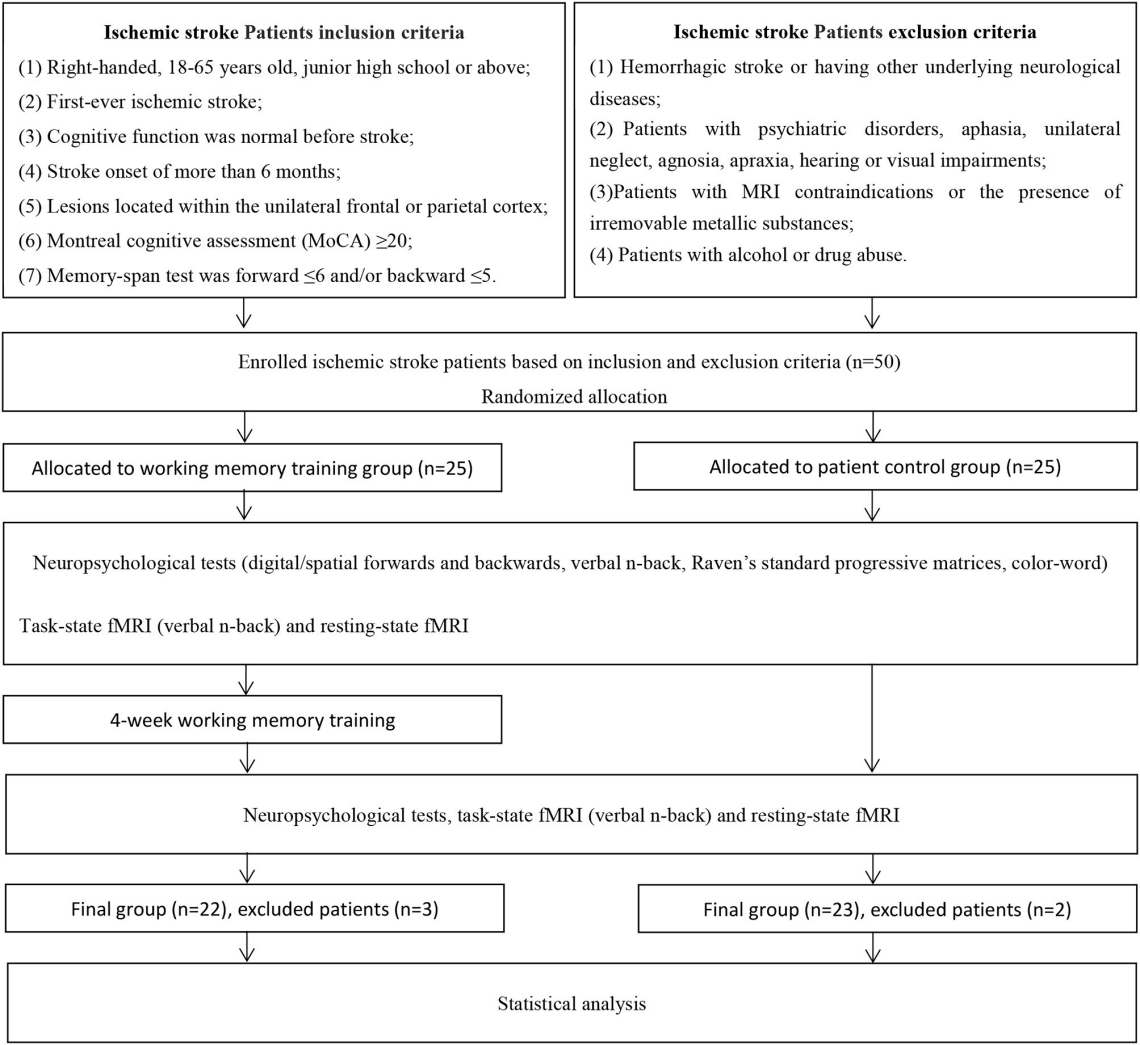
Flowchart of the screening and study protocol processes.

The current study was a parallel pre–post study, and a randomized, controlled, and single-blinded design was adopted. The enrolled patients were randomly assigned to a WMT group (*N* = 25) or a control group (CG) (*N* = 25). For the randomization of patients, random numbers were produced from a uniform distribution in the range 0–1, which were divided into two equal intervals, and each subject was assigned to the group corresponding to the sampled number. A qualified cognitive rehabilitation therapist and a neuropsychological evaluator who were responsible for training and evaluation were blinded to patient allocation at baseline and at the end of the experiment. A flowchart of the screening and study protocol processes can be seen in Figure 1.

### Procedures

Patients in the WMT group were instructed to commit to WMT for 4 weeks. Each training session lasted 30 min and took place twice a day, 5 days a week. The CG was a wait-list group; patients in CG were treated with the corresponding WMT after the end of the 4-week study cycle. All subjects were allowed to receive secondary prevention of ischemic stroke, including anti-platelet aggregation, statins for lipid regulation, blood pressure control, blood glucose control, diet, and lifestyle improvement. All patients were required to not take cognitive drugs or engage in other cognitive training during the study. All patients underwent neuropsychological tests, resting- and task-state fMRI assessments at baseline and after the 4-week intervention.

### Working memory training

In the WMT group, patients received WMT using the Working Memory Training System developed by the Rehabilitation Assessment Department within CRRC. Three training tasks were adopted, based on prior working memory training literature with some improvements (Lundqvist et al., 2010; Akerlund et al., 2013). Task A: A sequence of smiling faces appeared on the computer screen at any of eight locations. The subjects were required to determine whether the location of the currently presented smiling face was the same as that presented “n” items back. Task B: A series of playing cards appeared in the center of the screen one at a time. Participants were required to judge whether the currently presented stimulus was the same as the stimulus presented “n” items back. Task C: A clock was displayed in the middle of the screen with its hour and minute hands moving. Patients needed to determine whether the direction of the hour hand currently presented was the same as the hour hand presented “n” items back. The above n-back WM tasks contained three conditions (1-back, 2-back, and 3-back). Each block consisted of 20 trials, of which only 6 were targets. The stimulus was presented for 1,000 ms and the inter-stimulus interval was adjustable according to the ability of each patient (1,000 ms, 1,500 ms, 2,000 ms, 3,000 ms, 4,000 ms). All patients began with a 1-back task. If the patient made less than three errors in two consecutive blocks, the difficulty of “n” would increase by 1. Inversely, if the patient made six or more errors, the level of “n” would decrease by 1. Additionally, if the participant made more than five errors on a 1-back task, the inter-stimulus interval would increase.

### Neuropsychological tests

#### Digital forward and backward test

The aim of this test was to assess verbal WM. The test included number forward and number backward. If the patient could not remember a one-number range, a second test with the same number range was carried out. If they failed both assignments, then the test was stopped automatically. The digital reading speed was 1 s/word. The number of correctly memorized numbers was recorded.

#### Spatial forward and backward test

The aim of this test was to evaluate spatial WM. In a 4 × 4 matrix, the speed at which a square appeared was 1 s, and the patient recalled the position of squares one by one, including forward and backward. If the same number of square positions was misidentified twice, the test was stopped. The number of correctly memorized blocks was recorded.

#### N-back test

The n-back is one of the most frequently used tasks to measure individual WM capacity. The data were collected during fMRI scanning (see Task during fMRI below).

### Raven's standard progressive matrices (RSPM)

The RSPM test was used as a standardized intelligence task to measure fluid intelligence. Two forms of RSPM were designed. Each form consisted of 30 items and each item showed a 3 × 3 grid with the lower right corner missing. The participants had to choose the best from eight options to fill the missing location according to the rule of the matrix. Form A was made up of 1, 3, 5, 7,…,47, 49, while Form B consisted of 2, 4, 6, 8,…,58, 60. The two forms were equated for difficulty.

### Stroop color-word test (CWT)

Chinese characters in red and blue, as well as red and blue squares, were presented on the computer screen with red or blue color. The participants were required to press the left arrow key as soon as possible when the currently presented Chinese character or square was colored red, but press the right arrow key when the stimulus was presented in color blue. This test was applied to assess individual attention ability.

### Neuroimaging test

#### Image data acquisition

Images were scanned using a Siemens TrioTIM 3.0 Tesla MR system at the State Key Laboratory of Cognitive Neuroscience and Learning, Beijing Normal University. Both the resting- and task-state fMRI data were collected using an echo-planar imaging (EPI) sequence: repetition time (TR) = 2,000 ms, echo time (TE) = 30 ms, flip angle (FA) = 90°, field of view (FOV) = 200 × 200 mm, acquisition matrix = 64 × 64, number of slices = 33, voxel size = 3.0 × 3.0 × 3.0 mm3, and slice thickness/gap = 3/0 mm. The resting-state fMRI data were scanned before the task-state fMRI data. The T1-weighted anatomic images were obtained by using a 3D-spoiled gradient recalled (3D-SPGR) sequence: TR = 2,500 ms, TE = 3.5 ms, FA = 8°, voxel size = 1.0 × 1.0 × 1.0 mm3, acquisition matrix = 256 × 256, and number of slices = 144.

#### Task during fMRI

A block-design verbal n-back task was designed by using E-Prime (Psychology Software Tools, Sharpsburg, PA, USA) for a 1,000 ms duration with a 2,000 ms inter-stimulus interval. The task was presented with two conditions (1-back and 2-back), each condition included four blocks, and each block was made up of 10 continuous trials. A short break (30 s) between the blocks was given to allow subjects to rest. Stimulation materials were Chinese characters chosen randomly from word frequency, and if the current Chinese character shown on the screen matched the one presented “n” (*n* = 1 or 2) items back, subjects should press the button “Yes,” or else, “No.” The reaction times (RTs) and accuracy for each trial were recorded using E-Prime. Each time before scanning, a practice version with feedback (right or wrong) was provided for participants to practice until they understood the task.

#### Task-state fMRI analysis

A professional radiologist manually outlined the profiles of the lesions on T1-weighted images slice by slice using MRIcron (https://www.nitrc.org/projects/mricron), the images were then standardized to the MNI space and were resampled with a resolution of 1 × 1 × 1 mm^3^. The lesions were excluded in the following task- and resting-state fMRI analysis. Task-state fMRI data were preprocessed using the DPABI toolbox (Version 7.0, http://rfmri.org/dpabi) and SPM12 (http://www.fil.ion.ucl.ac.uk/spm/software/spm12) running under MATLAB R2016a (MathWorks, Inc., Natick, MA, USA). For each subject, the first 10 time points were abandoned to avoid transient signal changes before magnetization reached a steady state and to allow participants to acclimatize to the fMRI scanning noise. A least squares approach for slice timing was used to correct acquisition delay (slices = 33, reference slice was the last slice). After head motion correction, any participant with head motion exceeding 3 mm displacement in x, y, or z or 3° of angular motion was discarded. Then, the images were spatially normalized to T1 anatomic images (1 × 1 × 1 mm3) of each subject and resampled to 3 × 3 × 3 mm3. The smooth step with an isotropic Gaussian kernel of 6 mm was used to reduce the effects of poor normalization.

Subsequently, the smoothed functional images were entered into SPM12 for further processing. First level analysis (Specify 1st-level): For each patient, the smoothed images were analyzed at the voxel level using general linear models (GLMs) as implemented in SPM12. Each model contained a regressor with onset times for each condition (1-back and 2-back), which were convolved with a canonical hemodynamic response function. To further reduce any movement related to the task, the six movement parameters derived from the realignment stage were included as confounders in the model. To remove low-frequency noise, a high-pass filter was adopted. Contrast images for the blocked design conditions were generated. Second level analysis (Specify 2nd-level): The estimated parameter images were then entered into the second level group analysis. To explore pre–post activation changes, a paired *t*-test was performed within the WMT group and the CG. To investigate the influence of task load (task difficulty, 2-back > 1-back) on brain functional activation, a two-sample *t*-test was analyzed in the WMT group and the CG at baseline and after the 4-week cycle. To observe task-evoked functional activation at the group level, a one-sample *t*-test was performed. To examine between-groups functional activation differences at baseline, a two-sample *t*-test was adopted. Total lesion volume and lesion side were taken as covariates. To correct multiple comparisons, significance was determined at a voxel-level threshold (*p* < 0.001) and a cluster-level threshold (p < 0.05, FDR (false discovery rate) corrected).

#### Resting-state fMRI analysis

The resting-state fMRI data were preprocessed using CONN toolbox (Version 18b; http://www.nitrc.org/projects/conn) and SPM12 running under MATLAB R2016a with the following steps: (1) removal of initial scans (10 time points); (2) slice timing; (3) realignment (4) outlier detection (motion threshold: 3 mm; global signal threshold: Z = 9); (5) segmentation and normalization; (6)smoothing; (7) filter (0.01–0.08 Hz); (8) nuisance variable regression.

The seed-to-voxel method was adopted to calculate functional connectivity between seeds and the rest of the brain regions. According to the relevant literature (Christodoulou et al., 2001; Marvel and Desmond, 2010) and the present study, four brain regions that showed task-evoked brain activation in the 2-back condition at the baseline for the WMT group were chosen as seeds. Seed 1: left MFG [coordinates (−30, 51, 15)]; Seed 2: right MFG [coordinates (42, 33, 27)]; Seed 3: left inferior parietal lobule (IPL) [coordinates (−42, −39, 48)]; Seed 4: right IPL [coordinates (36, −48, 48)]. The seed area was made with the above four spatial coordinates and a radius of 5 mm. For each seed, we extracted the mean time series from the preprocessed resting-state functional images. Then, Pearson correlation coefficients between each seed and the rest of the brain voxels were calculated and converted to z scores using Fisher's r-to-z transformation. To explore pre–post functional connectivity changes, a paired *t*-test was performed within the WMT group. To correct multiple comparisons, significance was determined at a voxel-level threshold (*p* < 0.001) and a cluster-level threshold (*p* < 0.05, FDR correction, two-tailed).

### Statistical analysis

Between-groups comparisons of demographic, clinical features, and neuropsychological scales were analyzed using the Wilcoxon Mann-Whitney test for parametric continuous variables (means) and the chi-square test for categorical variables (proportions). Pre–post within-group comparisons of behavioral performance were performed using the Wilcoxon signed-rank test. All statistical analyses were performed using SPSS (v22.0) for Windows (SPSS Institute Inc., Chicago, IL, USA); a *p* < 0.05 was considered significant for all analyses.

## Results

### Behavioral results

Two patients in the CG withdrew from the study because of the recurrence of stroke. Two patients in the WMT group failed to complete the training procedure because of early discharge; the number of training days for them was 7 and 10, respectively. One patient in the WMT group was excluded because of severe head movement during fMRI scanning at baseline. The remaining 22 patients in the WMT group and 23 patients in the CG completed the experiment and underwent neuropsychological, resting- and task-state fMRI assessments at baseline and after the 4-week intervention. There was no significant difference between the two groups in demographic, clinical characteristics, and neuropsychological scales at baseline (all *p* > 0.05); see Table 1. Patients in the WMT group revealed significant improvements on tests of n-back (1-back and 2-back, both accuracy and mean RTs), memory-span (digital and spatial forward/backward), RSPM, and Stroop CWT (all p <0.05); see Table 2. Compared to the baseline, no significant behavioral improvement was found in the CG after the 4-week study cycle (all p > 0.05); see Table 2.

**Table 1:** Sample characteristics at baseline.

	**WMT group (*n =* 22)**	**CG (*n =* 23)**	***p*-value**
Age (years)	52.41 ± 7.58	51.22 ± 8.20	0.616
Gender (female/male)	7/15	9/14	0.608
Education (years)	16.55 ± 3.07	15.00 ± 3.06	0.112
Disease duration (months)	25.60 ± 17.91	32.09 ± 22.19	0.260
Hypertension	17 (77%)	15 (65%)	0.372
Diabetes	10 (45%)	13 (57%)	0.458
Dyslipidemia	12 (55%)	15 (65%)	0.465
Smoking	15 (68%)	12 (52%)	0.273
Lesion side (left %)	14 (64%)	10 (43%)	0.175
Frontal cortex lesion	12 (55%)	18 (78%)	0.092
Total lesion volume (cm^3^)	7.43 ± 4.13	5.63 ± 3.20	0.086
1-back (Accuracy, %)	83.07 ± 10.78	87.43 ± 10.35	0.172
1-back (mean RTs, ms)	888.00 ± 229.43	859.91 ± 281.05	0.716
2-back (Accuracy, %)	67.05 ± 13.11	71.47 ± 17.46	0.344
2-back (mean RTs, ms)	953.18 ± 266.86	900.47 ± 283.22	0.594
Digital forward	5.55 ± 1.34	5.78 ± 1.13	0.522
Digital backward	3.86 ± 0.77	3.52 ± 0.79	0.106
Spatial forward	3.86 ± 0.83	3.65 ± 0.93	0.476
Spatial backward	3.18 ± 0.59	3.22 ± 0.67	0.797
RSPM (max 30 items)	14.55 ± 4.64	16.13 ± 4.52	0.252
Stroop CWT (max 125)	102.55 ± 11.49	105.09 ± 12.42	0.480

WMT, working memory training; CG, control group; RTs, reaction times; RSPM, Raven's standard progressive matrices; CWT, color-word test.

**Table 2:** Changes in neurophysiological measures after intervention.

	**Group**	**Pre**	**Post**	**Difference**	***p*-value**
1-back (accuracy, %)	WMT	83.07 ± 10.78	93.15 ± 5.95	10.07 ± 7.90	<0.001
	CG	87.43 ± 10.35	88.16 ± 9.67	0.72 ± 3.47	0.328
1-back (RTs, ms)	WMT	888.00 ± 229.43	847.00 ± 207.45	−41.00 ± 70.89	0.049
	CG	859.91 ± 281.05	854.83 ± 269.04	−5.09 ± 63.35	0.704
2-back (Accuracy, %)	WMT	67.05 ± 13.11	72.44 ± 10.68	5.40 ± 10.44	0.024
	CG	71.47 ± 17.46	73.37 ± 14.13	1.90 ± 6.09	0.154
2-back (RTs, ms)	WMT	953.18 ± 266.86	851.73 ± 156.32	−101.45 ± 142.31	0.008
	CG	900.47 ± 283.22	878.22 ± 252.50	−22.26 ± 86.25	0.229
Digital forward	WMT	5.55 ± 1.34	6.18 ± 0.80	0.64 ± 0.90	0.006
	CG	5.78 ± 1.13	5.61 ± 0.99	−0.17 ± 1.19	0.404
Digital backward	WMT	3.86 ± 0.77	4.45 ± 0.67	0.59 ± 0.73	0.003
	CG	3.52 ± 0.79	3.78 ± 0.74	0.26 ± 0.75	0.109
Spatial forward	WMT	3.86 ± 0.83	4.32 ± 0.72	0.45 ± 0.67	0.008
	CG	3.65 ± 0.93	3.96 ± 0.82	0.30 ± 0.93	0.124
Spatial backward	WMT	3.18 ± 0.59	4.09 ± 0.92	0.91 ± 1.02	0.002
	CG	3.22 ± 0.67	3.43 ± 0.59	0.22 ± 0.67	0.132
RSPM	WMT	14.55 ± 4.64	16.06 ± 3.29	1.50 ± 2.43	0.009
	CG	16.13 ± 4.52	16.57 ± 4.19	0.43 ± 1.38	0.144
Stroop CWT	WMT	102.55 ± 11.49	108.68 ± 8.90	6.14 ± 6.31	<0.001
	CG	105.09 ± 12.42	104.22 ± 12.18	−0.87 ± 2.53	0.113

WMT, working memory training; CG, control group; RTs, reaction times; RSPM, Raven's standard progressive matrices; CWT, color-word test.

### fMRI results

**Figure 2:**
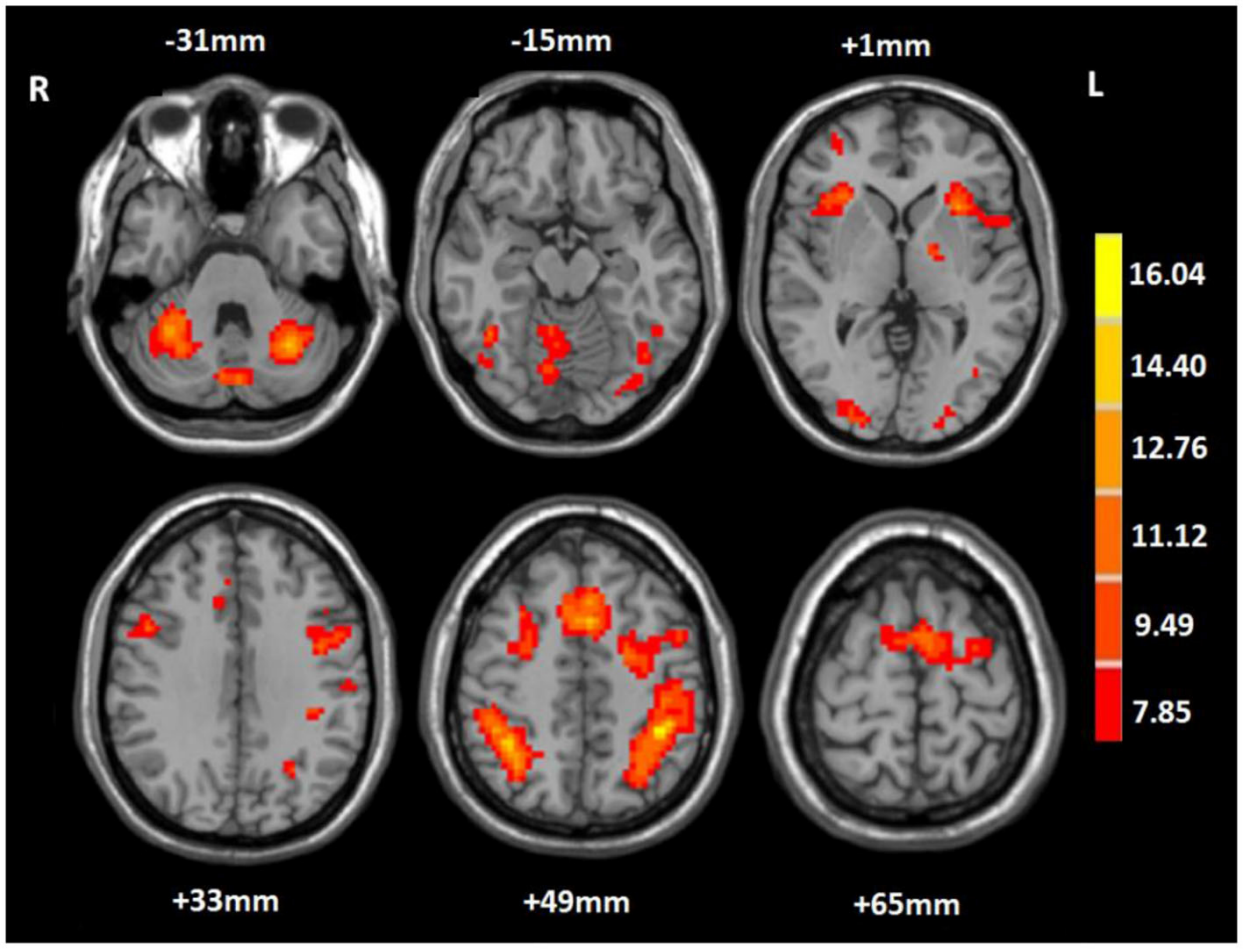
Mean task-related activation for the working memory training group in the 2-back condition at baseline. The red-yellow indicates positive activation (voxel-level *p* < 0.001; cluster-level *p* < 0.05, FDR corrected; cluster size ≥ 20 voxels). R, right; L, left.

In the 2-back condition at baseline, the WMT group showed significant task-evoked functional activation in the bilateral fronto-parietal and cerebellum, mainly including the bilateral middle frontal gyrus (MFG), IPL, and cerebellum posterior lobe (CPL), with a small amount of activation in the bilateral middle occipital gyrus (MOG) (voxel-level *p* < 0.001; cluster-level *p* < 0.05, FDR corrected; cluster size ≥ 20 voxels) (Table 3, Figure 2). The CG exhibited a similar task-evoked functional activation network to the WMT group in the 2-back condition at baseline; the two-sample *t*-test detected no significant difference in functional activation between the two groups at baseline (voxel-level *p* < 0.001; cluster-level *p* < 0.05, FDR corrected).

**Table 3:** Task-evoked brain functional activation in the 2-back condition at baseline for the WMT group.

**Brain regions**	**Voxel size**	**Peak MNI coordinates (x, y, z)**	**Peak *t*-value**
L MFG	1,480	−30, 51, 15	9.56
R MFG	95	42, 33, 27	10.64
R MFG	90	48, 9, 33	11.17
L IPL	426	−42, −39, 48	15.88
R IPL	377	36, −48, 48	15.31
L CPL	351	−30, −60,−30	14.82
R CPL	349	30,−60, 27	15.07
L MOG	30	−24, −90, 3	8.95
R MOG	92	24, −96, −3	12.45

WMT, working memory training; L, left; R, right; MNI, Montreal Neurological Institute; MFG, middle frontal gyrus; IPL, inferior parietal lobule; CPL, cerebellum posterior lobe; MOG, middle occipital gyrus.

**Figure 3:**
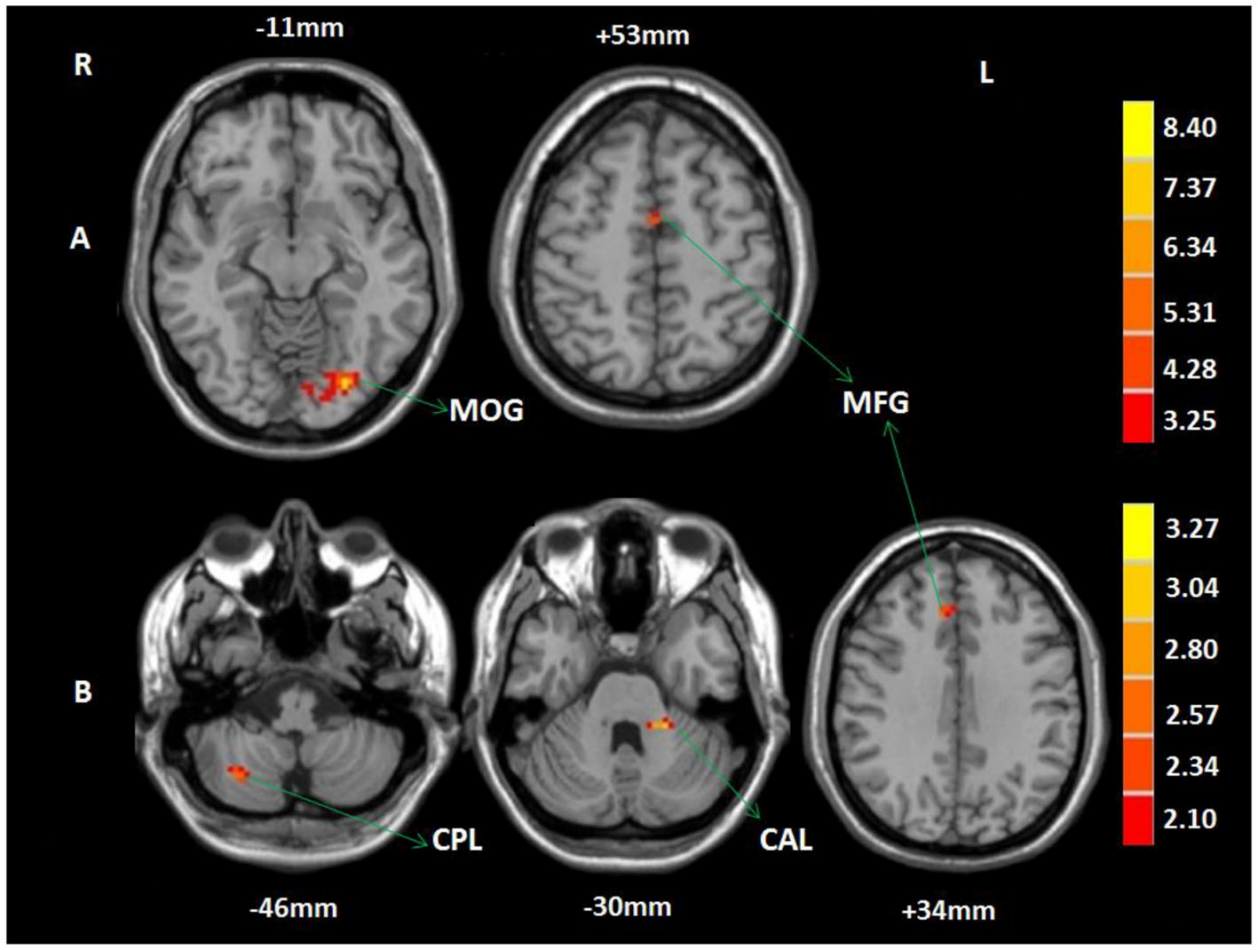
Training-related brain activation changes in the WMT group. **(A)** Brain regions showing increased activation in the 2-back condition after the intervention compared to baseline. **(B)** Brain regions showing increased activation in the 2-back > 1-back contrast after the intervention. The red-yellow indicates increased activation (voxel-level *p* < 0.001; cluster-level *p* < 0.05, FDR corrected). MOG, middle occipital gyrus; MFG, middle frontal gyrus; CPL, cerebellum posterior lobe; CAL, cerebellum anterior lobe; R, right; L, left.

In the 2-back condition, compared to baseline, patients in the WMT group showed increased functional activation in the left MFG and left MOG after the intervention; no decreased activation was observed (voxel-level *p* < 0.001; cluster-level *p* < 0.05, FDR corrected) (Table 4, Figure 3). In the 2-back condition, compared to baseline, patients in the CG showed no increased or decreased activation after the 4-week study cycle.

**Table 4:** Training-induced brain functional activation changes in the WMT group.

	**Brain regions**	**Peak MNI coordinates (x, y, z)**	**Voxel size**	**Peak *t*-value**
A	L MOG	−30, −81, −15	64	9.43
	L MFG	0, −3, 54	150	5.07
B	L CAL	−21, −39, −33	21	3.51
	R CPL	30, −66, −48	10	2.81
	R MFG	9, 30, 33	10	2.63

A, pre–post functional activation changes in 2-back condition; B, brain regions showing increased activation in the 2-back > 1-back contrast after the intervention; WMT, working memory training; L, left; R, right; MNI, Montreal Neurological Institute; MOG, middle occipital gyrus; MFG, middle frontal gyrus; CAL, cerebellum anterior lobe; CPL, cerebellum posterior lobe.

A two-sample *t*-test of the 2-back vs. the 1-back pattern of activation showed no increased or decreased activation in the CG at baseline and after the 4-week study cycle, or in the WMT group at baseline. However, in the WMT group, increased activation was observed in the left cerebellum anterior lobe (CAL), right CPL, and MFG in 2-back vs. 1-back contrast after intervention (voxel-level *p* < 0.001; cluster-level *p* < 0.05, FDR corrected) (Table 4, Figure 3).

**Figure 4:**
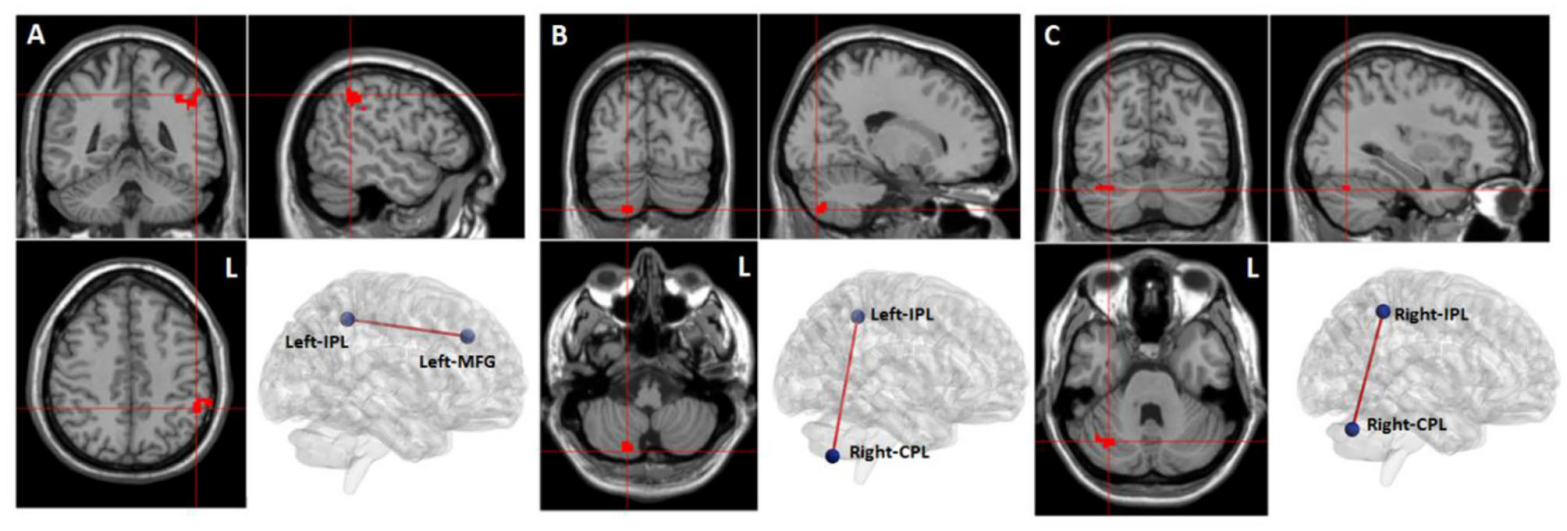
Training-related brain functional connectivity changes in the WMT group (voxel-level *p* < 0.001; cluster-level *p* < 0.05, FDR corrected, two-tailed). **(A)** Increased functional connectivity between the left middle frontal gyrus (MFG) and the left inferior parietal lobule (IPL) after training in the WMT group; **(B)** Increased functional connectivity between left IPL and right cerebellum posterior lobe (CPL) after training in the WMT group; **(C)** Increased functional connectivity between right IPL and right CPL.

Compared to the baseline in the WMT group, we observed increased resting-state functional connectivity between the left MFG and left IPL, between the left IPL and right CPL, and between the right IPL and right CPL after training (voxel-level *p* < 0.001; cluster-level *p* < 0.05, FDR corrected) (Table 5, Figure 4).

**Table 5:** Training-induced brain functional connectivity changes in the WMT group.

**Seeds**	**Brain regions**	**Peak MNI coordinates (x, y, z)**	**Voxel size**	**Peak *t*-value**
L MFG	L IPL	−51, −45, 45	67	8.62
L IPL	R CPL	18, −78, −48	16	8.52
R IPL	R CPL	36, −66, −30	12	8.27

MNI, Montreal Neurological Institute; L, left; R, right; MFG, middle frontal gyrus; IPL, inferior parietal lobule; CPL, cerebellum posterior lobe.

## Discussion

The neuropsychological results of a verbal n-back test that we used to examine the near-transfer effect of WMT revealed performance improvements in both 1-back and 2-back conditions. Patients in the WMT group made fewer mistakes and reacted quicker in an untrained verbal WM task 4 weeks after WMT. This kind of near-transfer effect was also reported in previous WMT studies (Vermeij et al., 2015; Hardy et al., 2021). The RSPM test used to assess the far-transfer effect of WMT indicated that training improved fluid intelligence (a non-verbal reasoning ability) in ischemic stroke patients in the WMT group. This finding was in agreement with previous studies that reported improved fluid intelligence after WMT (Jaeggi et al., 2010; Rudebeck et al., 2012). This improvement in fluid intelligence may be attributed to the training tasks that require the engagement of reasoning and executive processing, which are closely related to fluid intelligence. Our results also showed improvement in attention ability 4 weeks after the WMT in the WMT group. WM and attention are closely associated with each other; some previous studies have observed far-transfer effects on the tests of attention because tasks of WMT included regulation of attention and reduction of interference (Richmond et al., 2011; Brehmer et al., 2012).

Before training, neuroimaging results in the present study showed that verbal WM tasks evoked functional activation in a WM network, mainly including bilateral frontal-parietal and cerebellum regions, specifically, the bilateral MFG, IPL, and CPL. Our results were similar to previous fMRI studies that specifically investigated the brain areas involved in verbal WM (Paulesu et al., 1993; Chai et al., 2018), and were in keeping with previous studies that related specific lesion locations to WM performance in stroke patients (van Asselen et al., 2006; Baier et al., 2014).

Training can generate changes in brain functional activation modes. According to previous literature reports, training-induced brain activation modes can be grouped into the following four categories. First, is that the same brain regions are activated before and after training, but the activation intensity is increased in these regions after training (Westerberg and Klingberg, 2007). This “increase mode” may reflect an extensive recruitment of neural structures involved in the processing or the existing cortical neurons are more strongly responding to the task. Second, is that the same brain regions are activated at baseline and after training, but the activation intensity is decreased in these brain regions after intervention (Schneiders et al., 2011). This “decreased mode” may indicate the improved neural efficiency as a consequence of training, leaving more neurons for other task processing. The third mode is a combination of the above two modes, that is, the same brain regions are activated before and after training, but the activation is decreased in some of these brain regions while it is increased in other areas (Dahlin et al., 2008). This “redistribution mode” may suggest that training can lead to a quantitative shift in brain functional activation. The final mode is different from the other three modes. It is characterized by the activation of additional brain regions after training as compared to baseline (Opitz et al., 2014). This “network reorganization mode” may manifest that new neural routes are involved in the processing of tasks. After training, a similar brain network was activated during tasks of verbal WM in the WMT group, but WMT resulted in increased activation in the left MOG and MFG. This WMT-induced brain activation conforms to the “increase mode” described above. The increased activation in left MOG indicated increased compensatory ability to the impaired frontal-parietal regions and could be interpreted as more recruitment of neurons in the MOG into the WM network due to repetitive training. The increased activation in the left MFG might be attributed to enhanced neuronal responsiveness or the increased number of neurons within the left MFG that could result in an increase in regional fMRI signal underlying enhanced signal observed in the left MFG after intervention.

It was reported that healthy subjects maintained their ability to increase functional activation in the WM network with each increase in WM load (McAllister et al., 2001; Chen et al., 2012). We found that stroke patients in the current study were impaired in their abilities to elevate functional activation in the WM network as a response to the increasing WM load (2-back>1-back). This phenomenon suggested that with task difficulty increasing, no additional neurons within the WM network could be recruited to play compensatory roles for the completion of more difficult tasks. However, in the WMT group after training, increased activation was observed in the left CAL, right CPL, and MFG with increasing task difficulty. These results revealed that patients displayed improved abilities to increase activation in their WM network in response to the increasing WM load, and potentially indicated that WMT resulted in the left MFG and bilateral cerebellum regions being recruited to complete the challenging tasks.

After WMT, functional connectivity between the left MFG and left IPL, and between bilateral IPL and right CPL was increased. The enhancement of functional connectivity between these brain regions occurred within the WM network. In other words, WMT increased the resting-state functional connectivity within the WM network. It has been reported that the intensity of intrinsic resting-state activity in MFG and IPL is positively correlated with the intensity of functional activation in task-state activity (Zou et al., 2013), and, in general, the MFG and IPL are significantly activated in WM tasks. That is to say, the strength of the internal resting activity of MFG and IPL may be closely related to WM capacity. Deserno et al. (2012) reported that decreased functional connectivity between the MFG and IPL was closely related to the decline of WM capacity in patients with schizophrenia. In patients with acute stroke, it was suggested that impairment of WM was associated with damage to frontal-parietal regions (Martin et al., 2021). The MFG has been recognized to be crucial for maintaining information and supporting executive function during WM, especially high-load WM tasks (Volle et al., 2008; Bokde et al., 2010). The IPL was believed to play a critical role in maintaining phonological representations during phonological WM (Yue and Martin, 2022). It has been demonstrated that the cerebellum was included in the WM network and played a relevant role in subvocal rehearsal (Pleger and Timmann, 2018). It was also reported that cerebellum regions were responsible for executive control and for preventing irrelevant information from entering the WM network (Baier et al., 2014). However, the specific contribution of the cerebellum to the various processes involved in WM remains largely indistinct. As for why WMT led to resting-state functional connectivity changes within the WM network, we hypothesized that functional connectivity reflected past use, and those brain regions that were frequently used and activated simultaneously in the past have stronger functional connectivity. Thus, we suggested that the enhancement of resting-state functional connectivity within the WM network might be due to frequent and repetitive co-activation of these brain regions during WMT.

Several potential limitations involved in the present study should be considered. First, we did not assess the duration of training effects on behavior and brain as we did not set up a follow-up design. Furthermore, it is still not clear whether functional plasticity after WMT is based on structural plasticity, exploring structural changes after WMT in patients with stroke is therefore strongly recommended for subsequent studies. In addition, although comparisons of sample characteristics at baseline exhibited no statistical difference, the WMT group had larger mean lesion volumes and had more patients with left lesions than the CG, which might influence the outcomes more or less. Moreover, exploring the neural basis of the far-transfer effect following WMT in the future is desirable. Finally, future studies should compare the results of verbal WM and visual–spatial WM separately, as we only evaluated verbal WM during fMRI after WMT.

## Conclusion

Our study supported that WMT potentially improved WM capacity in ischemic stroke patients during the chronic stage, and that the training effects might transfer to fluid intelligence and attention ability. Our results also demonstrated that repeated WMT potentially increased brain activation and resting-state functional connectivity within the WM network in patients with ischemic stroke. These findings provided robust evidence supporting WMT as an effective intervention to enhance cognitive rehabilitation and also shed light on the functional neuroplasticity mechanism of WMT on cognitive recovery after ischemic stroke.

## Data availability statement

The original contributions presented in the study are included in the article/supplementary material, further inquiries can be directed to the corresponding authors.

## Ethics statement

The studies involving humans were approved by the Medical Ethics Committee of the CRRC. The studies were conducted in accordance with the local legislation and institutional requirements. The participants provided their written informed consent to participate in this study.

## Author contributions

ZC designed the study, performed the experiments, analyzed the data, and wrote the manuscript. XY did the financial support, review, and final approval of the article to be published. Both authors read and approved the final manuscript.
